# Independent and Combined Effects of Maternal Prepregnancy Body Mass Index and Gestational Weight Gain on Offspring Growth at 0–3 Years of Age

**DOI:** 10.1155/2016/4720785

**Published:** 2016-08-29

**Authors:** Wen-Yuan Jin, Yao Lv, Yu Bao, Li Tang, Zhi-Wei Zhu, Jie Shao, Zheng-Yan Zhao

**Affiliations:** ^1^Department of Children's Health Care, Children's Hospital, Zhejiang University School of Medicine, Hangzhou, Zhejiang 310003, China; ^2^Department of Neonatology, Children's Hospital, Zhejiang University School of Medicine, Hangzhou, Zhejiang 310003, China; ^3^Chinese Evidence-Based Center, West China Hospital, Sichuan University, Chengdu, Sichuan 610041, China

## Abstract

*Background*. The objective of this study was to investigate the independent and combined effects of maternal prepregnancy body mass index (BMI) and gestational weight gain (GWG) on offspring growth at 0–3 years old.* Methods*. A total of 826 pairs of nondiabetic mothers and their offspring were recruited in this study. Maternal information was abstracted from medical records and questionnaires. Offspring growth trajectories of weights and BMIs were depicted based on anthropometric measurements.* Results*. Offspring of mothers who were prepregnancy overweight/obese or obtained excessive GWGs continuously had greater weight and BMI *Z*-scores throughout the first 3 years of life. Children of prepregnancy overweight/obese mothers with excessive GWGs had a phenotype of higher weight and BMI *Z*-scores than those prepregnancy overweight/obese ones with nonexcessive GWGs from birth to 18 months. Maternal excessive GWGs increased offspring's risk of overweight/obesity at 12 months (AOR = 1.43, 95% CI: 1.03–2.00) and 24 months (AOR = 1.51, 95% CI: 1.02–2.25). Combination of excessive prepregnancy BMIs and GWGs was significantly associated with offspring's overweight/obesity at 30 months (AOR = 2.98, 95% CI: 1.36–6.53).* Conclusions*. Maternal prepregnancy overweight/obesity and excessive GWG are both significantly associated with rapid offspring growth from birth to 3 years old. Excessive GWGs strengthen the effects of high maternal prepregnancy BMIs on excessive offspring growth during their early life.

## 1. Introduction

Childhood overweight and obesity are an epidemic, worldwide public health problem with the prevalence increasing in a few decades. Previous studies have demonstrated predisposition for obese children to develop adiposity in adulthood accompanied with elevated susceptibility for type 2 diabetes, metabolic syndrome, and coronary heart diseases [1–6].

Maternal characteristics, including prepregnancy body mass index (BMI), gestational weight gain (GWG), and gestational diabetes mellitus (GDM), are significantly associated with short-term and long-term offspring growth. Maternal overweight/obesity prior to pregnancy and excessive weight gain during gestation have been regarded as potentially modifiable, independent risk factors for excessive fetal growth and childhood overweight/obesity, which are often connected with GDM or glucose intolerance [7–15]. Among the aforementioned three essential factors, prepregnancy overweight/obesity has been proposed as a genetic factor that shapes fetal and pediatric growth and leads to children's early onset and late onset overweight [1, 11–13, 15–18]. At the same time, GWG is becoming more and more noticeable for it can induce permanent alterations in fetal metabolism, which causes developmental programming of later life growth [19, 20]. Excessive GWG has been discovered to be associated with increased risk of GDM, cesarean section, large for gestational age (LGA), indicated preterm birth, and postpartum weight retention [21–24]; whereas inadequate GWG enhances the risk for small for gestational age (SGA), low birth weight, and spontaneous preterm birth [25, 26]. Apart from the independent impacts of two factors, recent evidence has implied that the combined effects of excessive maternal GWG and preconceptional obesity can result in higher neonatal birth weight, faster infancy weight gain, a sustained dysregulation of weight growth, and adverse childhood outcomes [1, 16].

By now, most of the existing studies evaluated the independent effects of maternal prepregnancy BMI or GWG on fetal growth, perinatal outcomes, and childhood obesity. In contrast, less studies explored their single and joint impacts on offspring growth patterns and consecutive growth trajectories during the first few years of life. This triggered our interests in conducting a clinical study among Chinese pregnant women and their offspring. Therefore, the present study was aimed at investigating the independent and combined effects of maternal prepregnancy BMI and GWG on offspring's growth and development in the first 3 years of life.

## 2. Methods

### 2.1. Study Subjects

From 1 January 2010 to 30 June 2011, late-stage pregnant women who attended regular prenatal examinations and intended to deliver babies in Yongkang, Huzhou, Cixi, Ninghai, Shaoxing, Yiwu, Dongyang, Wenling Maternal and Child Health Care Hospitals, and Women's Hospital, Zhejiang University School of Medicine, were invited to participate in our study. All the above hospitals were located in Zhejiang Province of China, receiving patients from urban and rural areas. Every participant signed the written informed consent before recruitment. At the same time, we obtained approval from Clinical Research Ethics Committee of the hospitals. The study groups were established on the basis of inclusion and exclusion criteria. Inclusion criteria for mothers were naturally conceived, singleton pregnancy, 28–37 weeks of gestation, having clear gestational age, and integrated medical records. Exclusion criteria for mothers were being conceived with assisted reproductive technology, multiple pregnancy, having diabetes, GDM, chromosomal abnormalities, inherited metabolic diseases or thyroid diseases, and experiencing serious infection during early pregnancy. All the mothers recruited were required to complete a questionnaire including maternal age, prepregnancy weight, lifestyle, and socioeconomic status. 30.6% of prepregnancy weights were derived from medical records and 69.4% were collected from questionnaires. Data of prepregnancy weights were reliable since the differences between first-trimester pregnancy weights (measured at the first prenatal visits within the first 12 weeks of pregnancy) and prepregnancy weights were very small. Details of height, previous obstetric history, and pregnancy complications like hypertensive disorders in pregnancy were collected from their medical records. They were followed up until delivery, and information about predelivery weight, delivery mode, gestational age, infant sex, birth weight, birth length, Apgar scores, and perinatal complications was recorded by obstetricians or midwives. Inclusion criteria for neonates were singleton and postpartum 5-minute Apgar scores ≥ 7. Exclusion criteria for neonates were congenital abnormalities, inherited metabolic diseases, and postpartum 5-minute Apgar scores < 7.

At the beginning, total of 1883 pregnant women were invited to participate in our study. There were 842 mothers excluded based on the above exclusion criteria (540 women had incomplete medical records, 168 subjects suffered from diabetes, GDM, thyroid diseases, or other special chronic diseases, and 134 cases were multiple pregnancies). After birth, 21 newborns were excluded because of congenital abnormalities or inherited metabolic diseases or postpartum 5-minute Apgar scores < 7. We included 1020 neonates at childbirth and 826 of them were followed up until 3 years old. Finally, 826 pairs of mothers and children were available for analysis.

### 2.2. Anthropometric Measurements and Feeding Pattern

Offspring in the present study were followed up and received regular physical examinations since birth, having their weights and lengths measured every 3 months in the first year of life and every 6 months in the second and third years. All the anthropometric data was collected by standardized measuring devices. Weight was measured to the nearest 0.01 kg with a digital scale (376, Hangzhou Seca Medical Measuring Apparatus Co., China). Length was measured to the nearest 0.1 cm with a length stadiometer (416, Hangzhou Seca Medical Measuring Apparatus Co., China). Both weight and length measurements were performed in light clothing. *Z*-scores of weight-for-age and BMI-for-age were calculated based on the World Health Organization (WHO) Child Growth Standards [27]. Moreover, when children received routine physical examinations, their parents were requested to finish a diet questionnaire regarding children's feeding pattern, quantity, and frequency.

### 2.3. Definitions

In our study, both maternal prepregnancy BMI and infant BMI were calculated with the formula: BMI = weight/(height^2^). Maternal prepregnancy BMIs were divided into underweight (<18.5 kg/m^2^), normal weight (18.5–24.9 kg/m^2^), overweight (25.0–29.9 kg/m^2^), and obese (≥30.0 kg/m^2^) groups according to the WHO BMI classification. Gestational weight gain was calculated through maternal predelivery weight minus prepregnancy weight. In accordance with the new recommendations issued by American Institute of Medicine (IOM), GWGs were categorized into adequate, inadequate, and excessive groups [28]. On the basis of prepregnancy BMIs, adequate GWGs were defined as 12.5–18.0 kg in underweight women, 11.5–16.0 kg in normal weight women, 7.0–11.5 kg in overweight women, and 5.0–9.0 kg in obese women. GWGs exceeding the above thresholds were defined as excessive, and those that fell below the thresholds were defined as inadequate.

According to Chinese Neonatal Birth Weight Curve for Different Gestational Age, neonates were stratified into appropriate/small/large for gestational age (AGA/SGA/LGA) [29]. AGA neonates were defined as birth weight that equalled or exceeded the 10th percentile and equalled or fell below the 90th percentile for gestational age. SGA/LGA neonates were defined as birth weight fell below the 10th percentile/exceeded the 90th percentile for gestational age. In addition, neonates were categorized into low birth weight (birth weight < 2,500 g), normal birth weight (birth weight = 2,500–4,000 g), and macrosomia (birth weight > 4,000 g) groups.

Feeding patterns were classified into exclusive breastfeeding, predominant breastfeeding, predominant formula feeding, and exclusive formula feeding. On the basis of WHO recommendations [30], exclusive breastfeeding was defined as giving infants no other food or drink, not even water, except breast milk (including milk expressed or from a wet nurse) for the first 6 months of life, but allowing the infants to receive oral rehydration salts, drops, and syrups (vitamins, minerals, and medicines). The definitions of childhood overweight and obesity were derived from the WHO Child Growth Standards [27]. Overweight was defined as a BMI ≥ 85 percentile and <95 percentile among the same age and same sex children. Obesity was defined as a BMI ≥ 95 percentile among the same age and same sex children.

### 2.4. Statistical Analysis

In our study, categorical variables were presented as *N* (%); normally distributed and skewed distributed continuous variables were, respectively, presented as mean (standard deviation, SD) and median (interquartile range, IQR). Chi-square test was applied in order to compare maternal and neonatal characteristics among different GWG groups at baseline. Kruskal-Wallis test was performed aiming at comparing offspring weight *Z*-scores and BMI *Z*-scores among different prepregnancy BMI classifications and GWG categorizations at each time point. We explored the associations between prepregnancy BMI status, GWG status, and childhood overweight/obesity at 1–3 years of age via multivariate logistic regression analysis, controlling for potential confounding variables. Maternal age at delivery, education background, family income, cigarette exposure, parity, delivery mode, offspring birth weight, sex, gestational age, and feeding pattern were regarded as confounders. We depicted offspring growth trajectories of weights and BMIs by age through GraphPad Prism 5. All the data analyses were performed with SPSS version 19.0 for Windows (SPSS Inc., Chicago, IL, USA). *p* values < 0.05 were considered as statistically significant.

## 3. Results

### 3.1. Maternal and Neonatal Characteristics at Baseline

Table 1 presents the maternal and neonatal characteristics of our study, according to the IOM GWG criteria. Based on these criteria, 8.6% (*n* = 71) mothers fell below, 58.1% (*n* = 480) met, and 33.3% (*n* = 275) exceeded the appropriate GWGs. Among the 826 eligible mothers, approximately 78% were 20–29 years old at delivery. The median (IQR) prepregnancy BMI was 20.03 (18.65–21.83) kg/m^2^. More than 64% of the women received junior college or higher education and 80% of them were nulliparous. Nearly 67% of the families had an annual income ranging from 7,500 to 30,000 US dollars. Approximately 28% of the mothers had the experience of cigarette exposure during pregnancy, including active smoking and passive smoking. The overall prevalence of cigarette exposure before and during pregnancy was raised to 35.6%. The morbidity of hypertensive disorders in pregnancy was 4.1%. About 54% of the neonates were boys and the mean (SD) birth weight was 3.38 (0.41) kg. On the basis of neonatal birth weight for gestational age categories, 84.5% of the neonates were AGA, 10.9% were LGA, and 4.6% were SGA. Expectant mothers who obtained excessive GWGs were more likely to be overweight/obese before pregnancy (*p* < 0.001), to experience cesarean section (*p* = 0.017), and to have macrosomia, LGA, or postterm offspring (*p* < 0.001). While pregnant women who gained inadequate GWGs were more likely to have low birth weight, SGA or premature offspring compared with those with adequate GWGs (*p* < 0.001).

### 3.2. Independent and Combined Effects of Maternal Prepregnancy BMI and GWG on Offspring Growth

**Figure 1:**
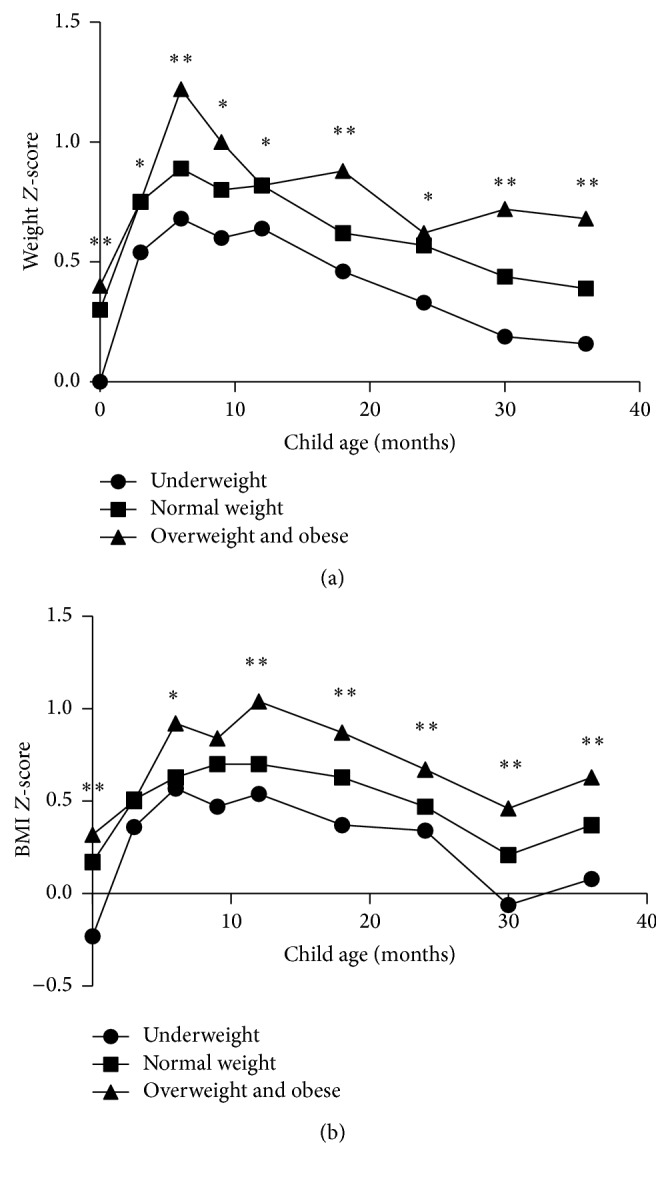
Offspring's weight *Z*-scores and BMI *Z*-scores associated with maternal prepregnancy BMI status at 0–3 years of age. ^*∗*^ *p* < 0.05, ^*∗∗*^ *p* < 0.01. Statistical analysis was performed with Kruskal-Wallis test.

Figure 1 displays offspring's weight *Z*-scores and BMI *Z*-scores associated with maternal prepregnancy BMI status at 0–3 years of age. According to our results, children of mothers who were overweight or obese before pregnancy were significantly heavier than those whose mothers stratified as prepregnancy normal weight or underweight from birth to 3 years old (*p* < 0.05). The differences were relatively attenuated at 3 months (*p* = 0.044), 12 months (*p* = 0.042), and 24 months (*p* = 0.026). Moreover, BMI *Z*-score trajectories showed that offspring of overweight or obese mothers were significantly larger compared with those of normal weight or underweight mothers during 0–3 years old (*p* < 0.05), except for 3 months (*p* = 0.154) and 9 months (*p* = 0.054).

**Figure 2:**
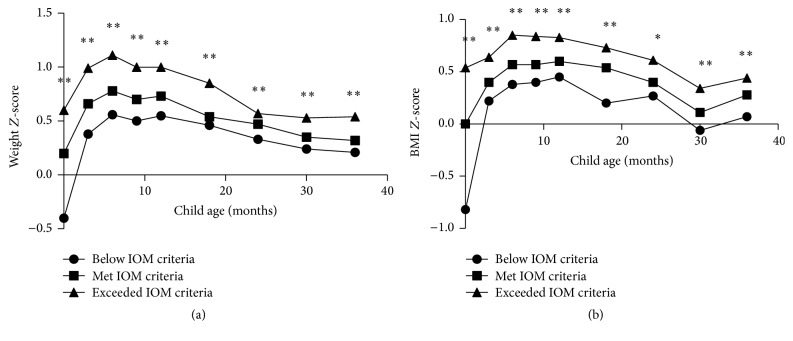
Offspring's weight *Z*-scores and BMI *Z*-scores associated with maternal gestational weight gain status at 0–3 years of age. ^*∗*^ *p* < 0.05, ^*∗∗*^ *p* < 0.01. Statistical analysis was performed with Kruskal-Wallis test.

Figure 2 reports offspring's weight *Z*-scores and BMI *Z*-scores associated with maternal GWG status at 0–3 years of age. Based on our results, offspring of mothers with excessive GWG tended to have higher weight *Z*-scores than those of mothers with inadequate or adequate GWG during 0–3 years old (*p* < 0.01). In addition, analysis for BMI *Z*-scores demonstrated that children of excessive GWG mothers were more likely to have higher BMIs at 0–3 years old (*p* < 0.05). Interestingly, we found that the discrepancies of weight *Z*-scores and BMI *Z*-scores among the three groups were attenuated since 24 months.

**Figure 3:**
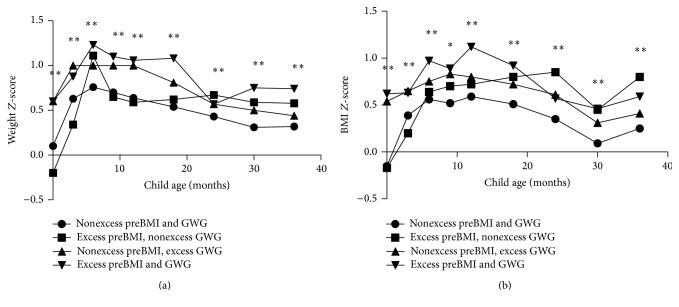
Offspring's weight *Z*-scores and BMI *Z*-scores associated with maternal prepregnancy BMI and gestational weight gain at 0–3 years of age. ^*∗*^ *p* < 0.05, ^*∗∗*^ *p* < 0.01. Statistical analysis was performed with Kruskal-Wallis test.

Figure 3 illustrates offspring's weight *Z*-scores and BMI *Z*-scores associated with maternal prepregnancy BMI and GWG status during ages 0–3 years. In our study, growth trajectories showed maternal prepregnancy overweight/obesity, excessive GWG, and prepregnancy overweight/obesity combined with excessive GWG; all led to persistent higher offspring BMI *Z*-scores in contrast with control groups since 9 months (*p* < 0.05). Furthermore, we found that prepregnancy overweight/obesity women were inclined to experience higher GWG, and 71.2% of overweight/obesity mothers exceeded the IOM criteria in our outcomes. Maternal excessive GWG amplified the effects of prepregnancy overweight/obesity on fetal and infant growth to some extent. Infants of mothers who were prepregnancy overweight/obese and obtained excessive GWG had a phenotype of higher BMI *Z*-scores than prepregnancy overweight/obese mothers with nonexcessive GWG from birth to 18 months. The differences reached statistical significance at birth (*p* = 0.002) and 3 months (*p* = 0.023). However, the joint effects of high prepregnancy BMI and high GWG on offspring BMI growth were attenuated since 24 months. Maternal prepregnancy BMI seemed to be a stronger determinant than the interaction of two factors at 24 and 36 months. Additionally, although offspring weight *Z*-scores may not possess the obvious trend as BMI *Z*-scores, a synergistic effect of the two factors on offspring growth could still be observed in Figure 3.

Table 2 shows the adjusted odds ratios (ORs) and 95% confidence intervals (95% CIs) for childhood overweight/obesity at 1–3 years of age associated with maternal prepregnancy BMI and GWG. Mothers with nonexcessive prepregnancy BMIs and GWGs and their offspring were regarded as the reference group. After adjustments for confounders, maternal excessive GWGs increased their offspring's risk of overweight/obesity at 12 months (*p* = 0.035, AOR = 1.43, 95% CI: 1.03–2.00) and 24 months (*p* = 0.041, AOR = 1.51, 95% CI: 1.02–2.25). Combination of maternal excessive prepregnancy BMIs and excessive GWGs was significantly associated with offspring's overweight/obesity at 30 months (*p* = 0.006, AOR = 2.98, 95% CI: 1.36–6.53). Furthermore, excessive prepregnancy BMIs were significantly associated with the occurrence of offspring overweight and obesity at 36 months (*p* = 0.006, AOR = 4.88, 95% CI: 1.62–14.52). In addition, we discovered that the effects of excessive prepregnancy BMIs on childhood overweight and obesity were continuously stronger than excessive GWGs at 1–3 years old. The joint influences of the two factors were stronger than the single ones at 12 and 18 months but gradually weakened since 24 months.

### 3.3. Associations between Feeding Pattern and Prepregnancy BMI, GWG Status, and Offspring's 1–3-Year-Old Overweight/Obesity

Table 3 depicts the associations between feeding pattern and prepregnancy BMI and GWG status and offspring's overweight/obesity at 1–3 years old. Children born to prepregnancy overweight/obesity mothers had higher probabilities to be exclusive formula fed (25.0%) compared with those born to underweight (12.3%) or normal weight (16.3%) mothers. Moreover, breastfeeding was associated with decreased risk for early childhood overweight and obesity. The morbidity of offspring's 1–3-year-old overweight/obesity in different feeding groups was, respectively, 39.9% (exclusive breastfeeding), 41.8% (predominantly breastfeeding), 52.3% (predominant formula feeding), and 54.3% (exclusive formula feeding).

## 4. Discussions

In this multicenter study, offspring of mothers with prepregnancy overweight/obesity or excessive GWGs always had greater weight and BMI *Z*-scores during the first 3 years of life. Excessive GWGs strengthened the effects of high maternal prepregnancy BMI on early life offspring growth and development. Children of mothers who were both prepregnancy overweight/obese and obtained excessive GWGs had a phenotype of higher BMI and weight *Z*-scores than those prepregnancy overweight/obese ones with nonexcessive GWGs before 24 months. The combination of maternal excessive prepregnancy BMIs and excessive GWGs was significantly associated with early childhood overweight/obesity.

Our study confirmed the importance of maternal prepregnancy BMI as a genetic factor in shaping offspring growth, consistent with previous studies [1, 11–13, 16–18]. We observed continuous greater weight and BMI *Z*-scores during 0–3 years of age in children of mothers who were overweight or obese before pregnancy. Moreover, we discovered that excessive maternal prepregnancy BMIs increased the risk for offspring adiposity. The underlying mechanism of this relationship may trace back to the fetal period. According to the majority of studies [31–34], fetal programming was the main contributor of excessive offspring growth and metabolic dysfunction. Maternal adiposity was associated with excessive production of reactive oxygen species and significant reduction of ATP levels in placenta, which impaired mitochondrial respiration and led to abnormal metabolic flexibility of trophoblasts [31]. Additionally, fetuses had developmental plasticity and responded to environmental factors via epigenetic modifications. For example,* Apolipoprotein D*, which was highly expressed in the central nervous system and essential for lipid regulation, was 9-fold upregulated in fetuses of obese pregnant women [32].

Except for maternal prepregnancy BMI, growth trajectories also showed that greater GWGs were significantly correlated with higher offspring weight *Z*-scores and BMI *Z*-scores from birth to 3 years old. In accordance with existing findings [7–9, 35, 36], excessive GWGs were significantly associated with excessive fetal growth (macrosomia and LGA). In addition, our results supported the fact that excessive GWGs is involved in fetal programming, which led to early childhood overweight and adiposity. Interestingly, we found the discrepancies of weight and BMI *Z*-scores among the inadequate, adequate, and excessive groups became attenuated after 24 months. Furthermore, logistic regression analysis implied that the effects (AOR and 95% CI) of excessive prepregnancy BMIs on childhood overweight and obesity were continuously stronger than excessive GWGs at 1–3 years old. These subtle differences suggested GWG was a potentially modifiable factor participating in the process of early childhood development. The effects of excessive GWGs on offspring growth patterns could be controlled if pregnant women had appropriate GWGs.

When turning to the interaction between maternal prepregnancy BMI and GWG, several previous studies [13, 37, 38] had investigated their combined effects on neonatal birth weights and BMIs. However, few explored their influences on offspring growth patterns or early childhood adiposity. Heerman et al. [1] discovered the joint impacts of prepregnancy overweight/obesity and excessive GWGs causing rapid infant growth throughout the first year of life. Leng et al. [15] believed the combination increased the risk for childhood overweight at 1–5 years old for those born to GDM mothers. Our study found that excessive GWGs strengthened the positive correlations between prepregnancy BMIs and offspring growth from birth to 18 months. Additionally, the combination of prepregnancy overweight/obesity and excessive GWGs was significantly associated with offspring's overweight/obesity at 30 months. Interestingly, the interaction was gradually attenuated since 24 months. In our opinion, the controversial outcome may be explained by the double effects of GWG. A recent review summarized the evidence from human and animal trials and proposed a U- or J-shaped association between maternal GWGs and childhood overweight/obesity, which meant both low GWGs and high GWGs could increase the risk for offspring overweight/obesity [39].

As we all know, growth and development are a complicated process influenced by multiple factors. Firstly, breastfeeding is an optimal source of nutrition for infants and an effective prevention against childhood obesity [40]. Our results reflected that exclusive breastfed infants consistently had lower BMI *Z*-scores from 9 months to 3 years old and decreased overweight/obesity prevalence at 1–3 years old compared with exclusive formula fed infants (39.9% versus 54.3%). In addition, catch-up growth is a common phenomenon occurring in premature offspring and children with poor intrauterine nutrition. They are more likely to experience accelerated weight gain in early life, which may lead to obesity and metabolic disorders [41, 42]. In our study, 20 neonates were premature and 38 neonates were SGA. Among these offspring who had a tendency of catch-up growth, 31.6% finally developed overweight or obesity at 1–3 years of age. Furthermore, the discrepancies of growth patterns between boys and girls were deserved to be taken into consideration. Although the overall growth trend was similar, a faster pattern of BMI growth could still be observed in boys from 9 months to 18 months (*p* < 0.05). At last, there are different approaches of defining childhood overweight/obesity in the world. Except for the WHO standards used in our study, International Obesity Task Force (IOTF) reference was another worldwide accepted criterion for childhood overweight/obesity [43]. We may draw different conclusions as a consequence of different standards.

The present study was a multicenter study with a follow-up that lasted for more than 3 years, collecting a series of data from both mothers and children. We recorded offspring weights and BMIs from birth to 3 years old, which provided consecutive growth trajectories of them. Not only have we evaluated the independent effects of maternal prepregnancy BMIs/GWGs on offspring weight *Z*-scores and BMI *Z*-scores, but also we have explored their combined impacts on growth patterns in early childhood. Moreover, we investigated the associations between excessive prepregnancy BMI, excessive GWG, and childhood overweight/obesity at 1–3 years of age. The interaction between two maternal factors was the main attractive novel point of our study. Additionally, GDM mothers had not been enrolled at baseline, which excluded the confounding interference of GDM on excessive offspring growth. Thus, we extensively discussed the importance of interventions before pregnancy and in utero periods for preventing pediatric obesity.

There are still some limitations in our study. First of all, our conclusions were subjected to the limited sample size and loss to follow-up bias. Large-sample based and well-responded studies will be more representative to further clarify the joint effects of the two factors on offspring growth in Chinese population. Secondly, approximately 70% of maternal prepregnancy weights were self-reported in the data collection, so there exist the possibilities of recall bias. In addition, apart from weights and BMIs, several anthropometric measurements and biomarkers can reflect children's development, including waist circumference, subcutaneous adipose tissue, visceral adipose tissue, high-density lipoprotein cholesterol, and triglycerides [16]. We failed to take them into consideration when evaluating offspring growth patterns and childhood obesity. Moreover, we cannot rule out the possibility of residual confounding. Dietary intake is an important contributor that shapes offspring's growth and development, but we failed to include enough relevant information for further comparison.

## 5. Conclusions

Our findings suggested that maternal prepregnancy overweight/obesity and excessive GWGs were continuously associated with rapid offspring growth throughout the first 3 years of life. Excessive GWGs strengthen the effects of high maternal prepregnancy BMI on excessive offspring growth and development during their early life. The present study emphasized the importance of preconception and prenatal interventions for improving pregnancy outcomes and preventing pediatric obesity.

## Figures and Tables

**Table 1:** Maternal and neonatal characteristics at baseline.

Characteristics	Below IOM criteria (*n* = 71)	Met IOM criteria (*n* = 480)	Exceeded IOM criteria (*n* = 275)	*p*
*Maternal characteristics*				
Maternal age at delivery (year)				0.246
20–29	51 (71.8)	381 (79.4)	214 (77.8)	
30–34	15 (21.1)	85 (17.7)	46 (16.7)	
≥35	5 (7.0)	14 (2.9)	15 (5.5)	
Prepregnancy body mass index				<0.001^*∗∗*^
Underweight	26 (36.6)	131 (27.3)	33 (12.0)	
Normal weight	45 (63.4)	334 (69.6)	205 (74.5)	
Overweight and obese	0 (0)	15 (3.1)	37 (13.5)	
Maternal education				0.156
≥University	19 (26.8)	156 (32.5)	108 (39.3)	
Junior college	30 (42.3)	141 (29.4)	77 (28.0)	
High school	11 (15.5)	101 (21.0)	51 (18.5)	
Junior school	11 (15.5)	77 (16.0)	33 (12.0)	
≤Primary school	0 (0)	3 (0.6)	3 (1.1)	
Unknown	0 (0)	2 (0.4)	3 (1.1)	
Family income (US dollars/year)				0.282
>30,000	2 (2.8)	26 (5.4)	9 (3.3)	
15,000–30,000	28 (39.4)	167 (34.8)	76 (27.6)	
7,500–15,000	22 (31.0)	149 (31.0)	105 (38.2)	
3,000–7,500	13 (18.3)	101 (21.0)	62 (22.5)	
<3,000	6 (8.5)	32 (6.7)	17 (6.2)	
Unknown	0 (0)	5 (1.0)	6 (2.2)	
Cigarette exposure in pregnancy				0.937
No	51 (71.8)	342 (71.3)	198 (72.0)	
Yes	20 (28.2)	136 (28.3)	75 (27.3)	
Unknown	0 (0)	2 (0.4)	2 (0.7)	
Cigarette exposure overall				0.936
No	45 (63.4)	309 (64.4)	174 (63.3)	
Yes	26 (36.6)	169 (35.2)	99 (36.0)	
Unknown	0 (0)	2 (0.4)	2 (0.7)	
Hypertensive disorders in pregnancy				0.569
No	69 (97.2)	462 (96.3)	261 (94.9)	
Yes	2 (2.8)	18 (3.8)	14 (5.1)	
Parity				0.878
Primipara	58 (81.7)	382 (79.6)	222 (80.7)	
Multipara	13 (18.3)	98 (20.4)	53 (19.3)	
Delivery mode				0.017^*∗*^
Vaginal delivery	27 (38.0)	216 (45.0)	95 (34.5)	
Cesarean section	44 (62.0)	264 (55.0)	180 (65.5)	
*Neonatal characteristics*				
Infant sex				0.784
Male	36 (50.7)	259 (54.0)	152 (55.3)	
Female	35 (49.3)	221 (46.0)	123 (44.7)	
Gestational age (GA) (week)				<0.001^*∗∗*^
Premature (GA < 37)	8 (11.3)	8 (1.7)	4 (1.5)	
Full term (37 ≤ GA < 42)	63 (88.7)	472 (98.3)	268 (97.5)	
Postterm (GA ≥ 42)	0 (0.0)	0 (0)	3 (1.1)	
Birth weight (g)				<0.001^*∗∗*^
<2500	6 (8.5)	6 (1.3)	0 (0)	
2500–4000	65 (91.5)	459 (95.6)	246 (89.5)	
>4000	0 (0)	15 (3.1)	29 (10.5)	
Weight for gestational age				<0.001^*∗∗*^
SGA	12 (16.9)	23 (4.8)	3 (1.1)	
AGA	57 (80.3)	425 (88.5)	216 (78.5)	
LGA	2 (2.8)	32 (6.7)	56 (20.4)	

IOM: American Institute of Medicine; GA: gestational age; SGA/AGA/LGA: small/appropriate/large for gestational age.

Maternal and neonatal characteristics were presented as *N* (%). Statistical analysis was performed by Chi-square test.

^*∗*^ *p* < 0.05, ^*∗∗*^ *p* < 0.01.

**Table 2:** Associations between maternal prepregnancy body mass index, gestational weight gain, and offspring overweight/obesity at 1–3 years old.

Child age	NN	EN		NE		EE	
	OR	AOR (95% CI)	*p*	AOR (95% CI)	*p*	AOR (95% CI)	*p*
12 months	1.00 (reference)	1.56 (0.53–4.58)	0.420	1.43 (1.03–2.00)	0.035^*∗*^	1.74 (0.84–3.59)	0.134
18 months	1.00 (reference)	1.67 (0.57–4.94)	0.350	1.36 (0.97–1.92)	0.078	1.75 (0.85–3.62)	0.130
24 months	1.00 (reference)	2.62 (0.81–8.46)	0.108	1.51 (1.02–2.25)	0.041^*∗*^	1.87 (0.84–4.18)	0.125
30 months	1.00 (reference)	3.66 (0.90–14.86)	0.069	1.46 (0.96–2.24)	0.080	2.98 (1.36–6.53)	0.006^*∗∗*^
36 months	1.00 (reference)	4.88 (1.62–14.52)	0.006^*∗∗*^	1.44 (0.96–2.18)	0.080	2.01 (0.89–4.53)	0.093

BMI: body mass index; GWG: gestational weight gain; AOR: adjusted odds ratio; CI: confidence interval.

Group NN: nonexcessive prepregnancy BMI and nonexcessive GWG.

Group EN: excessive prepregnancy BMI and nonexcessive GWG.

Group NE: nonexcessive prepregnancy BMI and excessive GWG.

Group EE: excessive prepregnancy BMI and excessive GWG.

Adjusted ORs were corrected for maternal age, education background, family income, cigarette exposure, parity, delivery mode, offspring birth weight, sex, gestational age, and feeding pattern.

^*∗*^ *p* < 0.05, ^*∗∗*^ *p* < 0.01.

**Table 3:** Associations between feeding pattern and prepregnancy body mass index, gestational weight gain status, and offspring's 1–3-year-old overweight/obesity.

Maternal and offspring factors	Exclusive breastfeeding	Predominant breastfeeding	Predominant formula feeding	Exclusive formula feeding
*Prepregnancy BMI status (%)*				
Underweight	21.4	40.8	25.5	12.3
Normal weight	24.2	36.8	22.6	16.3
Overweight and obese	23.1	38.5	13.5	25.0
*GWG status (%) *				
Below IOM criteria	21.7	38.3	24.4	15.6
Met IOM criteria	16.9	43.7	31.0	8.5
Exceeded IOM criteria	24.4	41.1	21.8	12.7
*Offspring overweight and obesity (%)*				
1–3 years old	39.9	41.8	52.3	54.3

BMI: body mass index; GWG: gestational weight gain; IOM: American Institute of Medicine.
